# Development of TEM-1 β-lactamase based protein translocation assay for identification of *Anaplasma phagocytophilum* type IV secretion system effector proteins

**DOI:** 10.1038/s41598-019-40682-8

**Published:** 2019-03-12

**Authors:** Jiafeng Zhu, Meiling He, Wenting Xu, Yuanyuan Li, Rui Huang, Shuyan Wu, Hua Niu

**Affiliations:** Department of Microbiology, College of Medicine, Soochow University, Suzhou, Jiangsu Province 215123 China

## Abstract

*Anaplasma phagocytophilum*, the aetiologic agent of human granulocytic anaplasmosis (HGA) is an obligate intracellular Gram-negative bacterium with the genome size of 1.47 megabases. The intracellular life style and small size of genome suggest that *A. phagocytophilum* has to modulate a multitude of host cell physiological processes to facilitate its replication. One strategy employed by *A. phagocytophilum* is through its type IV secretion system (T4SS), which translocates bacterial effectors into target cells to disrupt normal cellular activities. In this study we developed a TEM-1 β-lactamase based protein translocation assay and applied this assay for identification of *A. phagocytophilum* T4SS effectors. An *A. phagocytophilum* hypothetical protein, APH0215 is identified as a T4SS effector protein and found interacting with *trans*-Golgi network in transfected cells. Hereby, this protein translocation assay developed in this study will facilitate the identification of *A. phagocytophilum* T4SS effectors and elucidation of HGA pathogenesis.

## Introduction

Bacterial type IV secretion system (T4SS) is a translocation apparatus which transports macromolecules, such as DNA and protein substrates from bacteria to prokaryotic and eukaryotic cells, facilitating dissemination of mobile genetic elements, or dysregulating physiological processes of host cells, resulting in disease development^1^. Based on genetic, biochemical, and structural studies, T4SS generally are classified into two types, T4ASS and T4BSS^2,3^. Shared among bacterial P- and F-type conjugation systems, as well as *Agrobacterium tumefaciens* VirB/VirD4 system, T4ASS transporter is composed of functionally distinct modules including pilus, outer membrane core complex (OMCC), inner membrane complex (IMC) and type IV coupling protein (T4CP)^1,2^. These modules are assembled with a conserved set of approximately 11 VirB proteins from VirB1 to VirB11, and VirD4 subunit, among of which VirD4 is a T4CP^2^. In a macromolecule translocation model, VirD4 first recognizes its cognate substrates and deliver them to IMC (core components: VirB3, VirB6, VirB8), followed by the transfer to OMCC (core components:VirB7, VirB9, VirB10) and pilus (core components:VirB2, VirB5)^2^. VirD4 is composed of N-terminal transmembrane domain (NTD), and cytoplasmic domain which contains conserved nucleotide binding domain (NBD) and a sequence-variable α-helical bundle termed the all-alpha-domain (AAD), and in most cases a sequence-variable C-terminal domain (CTD)^4,5^. NTD acts for cytoplasmic membrane anchoring and participates the interaction with other VirB proteins, while AAD and CTD function in recognition, binding and recruitment of substrates^6–8^. Although there is highly specific interaction between T4CP and its cognate substrates, the interaction between the T4CP and the T4SS is less specific: a single T4CP can interact functionally with several heterologous conjugative T4SS^9^.

Human granulocytic anaplasmosis (HGA) caused by Gram-negative obligate intracellular rickettsial bacterium, *Anaplasma phagocytophilum* is a nonspecific febrile disease clinically manifestated with fever, malaise, headache, myalgia, and arthralgia. Leukopenia, thrombocytopenia, and elevations in serum hepatic aminotransferases are frequently found in HGA patients by laboratory tests^10,11^. *A. phagocytophilum* primarily invades first-line immune defensive cells, neutrophils, and replicates in membrane-bound inclusions in the cytoplasm of host cells^12^. To complete its intracellular life cycle, *A. phagocytophilum* alters a multitude of physiological activities of host cells, such as apoptosis inhibition, cytoskeleton remodeling, inhibition of innate immune response, induction of autophagosome formation, and interception of vesicle trafficking^12–23^. Although several *A. phagocytophilum* proteins were identified to participate in certain specific changes, such as AnkA, Ats-1, AptA and UMPK^16,18,20,24–27^, the bacterial factors involved in the regulation of other physiological activities are largely unknown. Among these identified *A. phagocytophilum* proteins, AnkA and Ats-1, as wells as HGE14 are the T4SS effectors^28^. As the intracellular bacteria, *Legionella pneumophila* and *Coxiella burnetii* regulate multitudes of host cellular activities by translocating hundreds of effectors into host cells. Similarly, to adapt the obligate intracellular life style, *A. phagocytophilum* may translocate more effectors than currently identified. Due to the obligate intracellular life style, the methods for genetic manipulation of *A. phagocytophilum* are limited. The identification of *A. phagocytophilum* T4SS effectors were routinely performed by using surrogate hosts, including *A. tumefaciens*, *L. pneumophila*, *C. burnetii* and *Escherichia coli*, together with indication systems using Cre-*lox*P recombination and enzyme activity of adenylate cyclase^8,16,28,29^. However, the widely used reporter, TEM-1 β-lactamase has not been applied to *A. phagocytophilum* T4SS studies, although it has been employed for long time in applications such as mammalian gene expression assay, and bacterial effector protein translocation assay in eukaryotic cells^30,31^. In this study we develop a TEM-1 β-lactamase based protein translocation reporter system to provide a simple, flexible assay to identify *A. phagocytophilum* T4SS effectors. Furthermore, by application of this assay and immunofluorescence labeling, an *A. phagocytophilum* hypothetical protein, APH0215 is identified as a T4SS effector protein and found interacting with *trans*-Golgi network in transfected cells.

## Results

### Development of a TEM-1 β-lactamase based protein translocation assay for *A. phagocytophilum* T4SS

**Figure 1:**
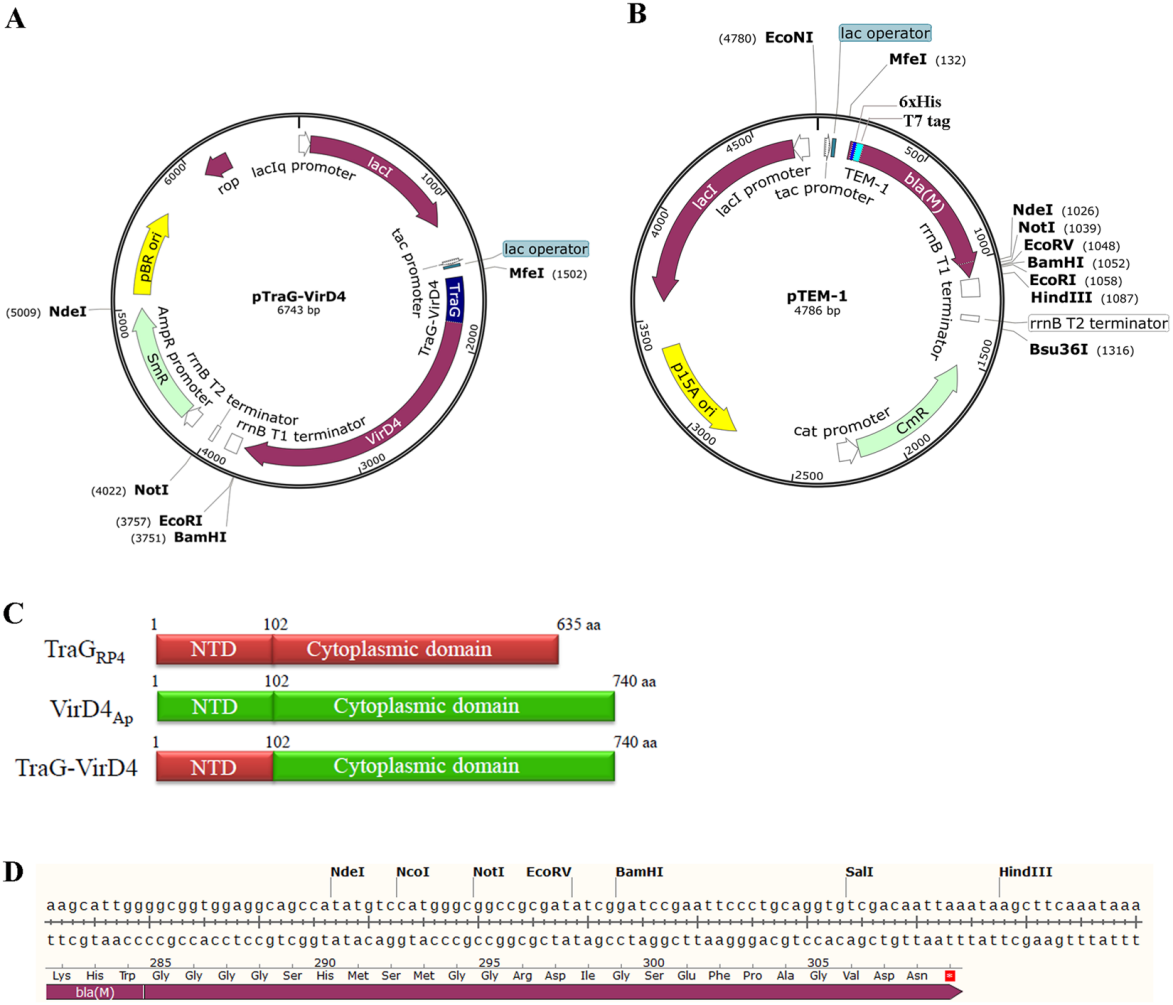
Construction of plasmids pTraG-VirD4 and pTEM-1. (**A**) pTraG-VirD4 map. The synthesized DNA fragment encoding chimeric T4CP, TraG-VirD4 was cloned into the pMal-c5X between MfeI and BamHI sites, followed by the antibiotic resistance gene swapping from ampicillin resistance gene (Amp^R^) to Streptomycin resistance gene (Sm^R^), leading to the generation of pTraG-VirD4 with the length of 6743 base pairs (bp). The expression of TraG-VirD4 is under control of tac promoter. (**B**) pTEM-1 map. The synthesized DNA fragment including tac promoter, 6xHistidine, T7 tag, TEM-1(bla(M))-encoding sequences, multiple cloning sites, and transcription terminator (rrnB T1 and rrnB T2) was cloned into plasmid pACYCDuet-1 between EcoNI and Bsu36I sites, leading to the generation of pTEM-1 with the length of 4786 bp. The expression of TEM-1 fused with 6 × His and T7 tags is under control of tac promoter. (**C**) The schematic diagram of TraG of RP4 plasmid (TraG_RP4_, molecular mass:69.9 kDa), VirD4 of *A. phagocytophilum* T4SS (VirD4_Ap_, molecular mass:84.9 kDa), and the chimeric T4CP (TraG-VirD4, molecular mass: 84.2 kDa). TraG-VirD4 was constructed by fusion of the N-terminal transmembrane domain (NTD) of TraG_RP4_ (1^st^–102^nd^ amino acids (aa)) with the cytoplasmic domain of VirD4 of *A. phagocytophilum* (103^rd^–740^th^ amino acids, molecular mass: 73.1 kDa), which contains nucleotide binding domain, all-alpha-domain, and C-terminal domain. (**D**) The multiple cloning sites in pTEM-1. The DNA sequence is showed in top with endonuclease cleavage sites, while the deduced amino acid sequence is presented in bottom, including partial TEM-1 (bla(M)) encoding sequence. *Stop codon.

**Figure 2:**
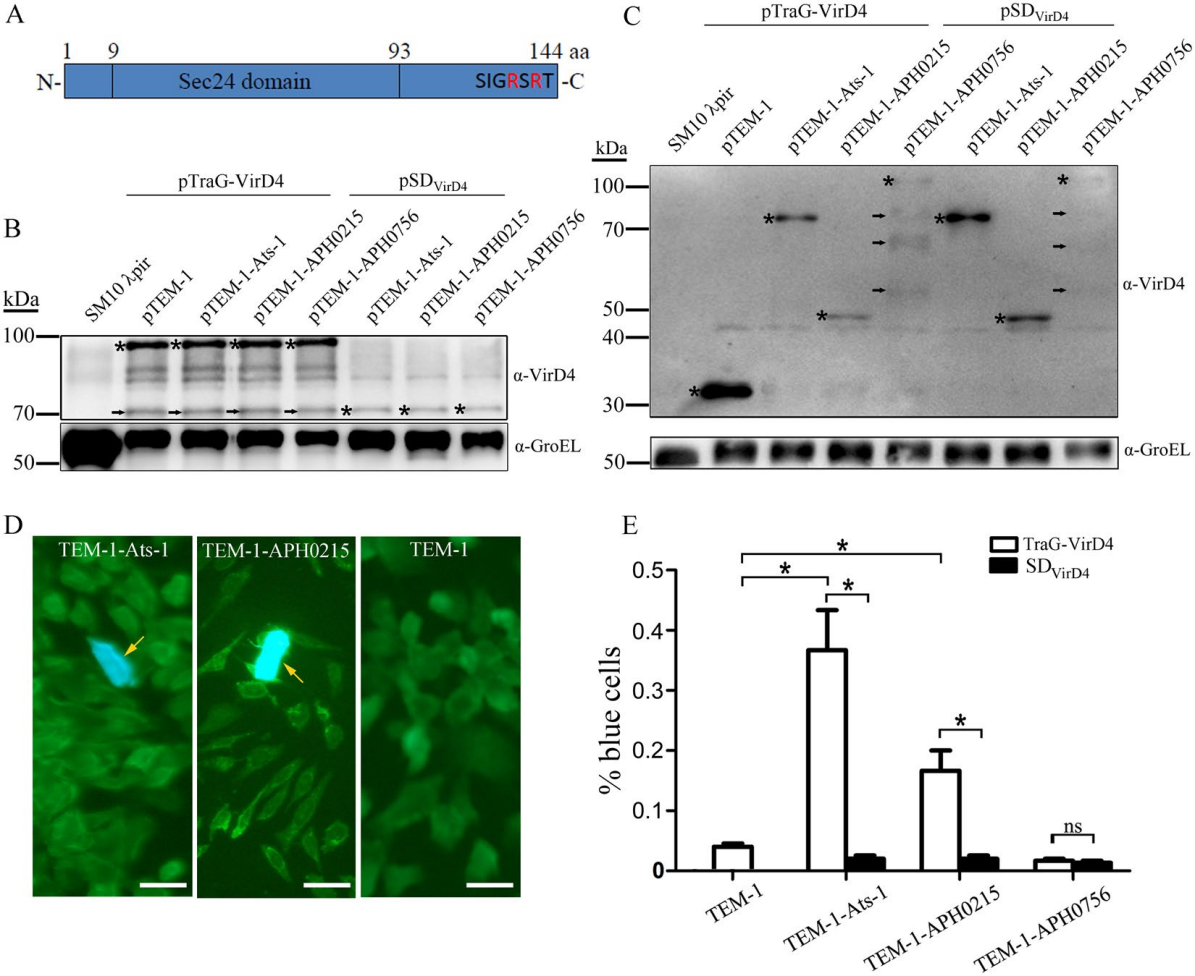
TEM-1 β-lactamase based protein translocation assay. (**A**) The schematic diagram of APH0125. APH0215 contains a Sec24 domain (9^th^–93^rd^ aa), and positively charged Arginine (R) at its C-terminus. (**B**) Western blot analysis for TraG-VirD4 and SD_VirD4_ expression, probed with rabbit anti-VirD4 antibody, in SM10 λpir cotransformed with plasmid pairs pTraG-VirD4 and pTEM-1, pTraG-VirD4 and pTEM-1-Ats-1, pTraG-VirD4 and pTEM-1-APH0215, pTraG-VirD4 and pTEM-1-APH0756, pSD_VirD4_ and pTEM-1-Ats-1, pSD_VirD4_ and pTEM-1-APH0215, or pSD_VirD4_ and pTEM-1-APH0756. Bacterial cultures were harvested after 2 h induction with IPTG, and before incubation with CHO K1 cells. Original SM10 λpir (SM10 λpir) is used as negative control. Asterisks highlight the expected bands. Arrows indicate the degraded product of TraG-VirD4. GroEL bands, probed by anti-GroEL antibody were used to normalize the loading amount of bacterial lysates. (**C**) Western blot analysis for TEM-1, TEM-1-Ats-1, TEM-1-APH0215, and TEM-1-APH0756 expression, probed with mouse monoclonal anti-T7 tag antibody, in SM10 λpir cotransformed with plasmid pairs pTraG-VirD4 and pTEM-1, pTraG-VirD4 and pTEM-1-Ats-1, pTraG-VirD4 and pTEM-1-APH0215, pTraG-VirD4 and pTEM-1-APH0756, pSD_VirD4_ and pTEM-1-Ats-1, pSD_VirD4_ and pTEM-1-APH0215, or pSD_VirD4_ and pTEM-1-APH0756. Bacterial cultures were harvested after 2 h induction with IPTG, and before incubation with CHO K1 cells. Original SM10 λpir (SM10 λpir) is used as negative control. Asterisks highlight the expected bands. Arrows indicate the degraded products of TEM-1-APH0756. GroEL bands, probed by anti-GroEL antibody were used to normalize the loading amount of bacterial lysates. (**D**) Fluorescence signal conversion in CHO K1 cells incubated with SM10 λpir cotransformed with plasmid pairs pTraG-VirD4 and pTEM-1-Ats-1 (TEM-1-Ats-1), or pTraG-VirD4 and pTEM-1-APH0215 (TEM-1-APH0215), pTraG-VirD4 and pTEM-1 (TEM-1). CHO K1 cells emitting blue fluorescence are highlighted with arrows. Scale bars: 20 μm. (**E**) The percentage of blue cells in CHO K1 cells after incubation with SM10 λpir cotransformed with plasmid pairs pTraG-VirD4 and pTEM-1 (TEM-1), pTraG-VirD4 and pTEM-1-Ats-1 (TEM-1-Ats-1), pTraG-VirD4 and pTEM-1-APH0215 (TEM-1-APH0215), pTraG-VirD4 and pTEM-1-APH0756 (TEM-1-APH0756), pSD_VirD4_ and pTEM-1-Ats-1 (TEM-1-Ats-1), pSD_VirD4_ and pTEM-1-APH0215 (TEM-1-APH0215), or pSD_VirD4_ and pTEM-1-APH0756 (TEM-1-APH0756). *: Significant difference (*P* < 0.01) between groups indicated with lines by Student *t*-test. ns: no significant difference between groups. Data are presented as the means and standard deviations of samples from three independent experiments. The full-length blots for all cropped ones are included in Supplemental Fig. S1 respectively.

T4CP specifically interacts with its cognate substrates through its AAD domain, meanwhile it can interact functionally with heterologous T4SS. Based on this observation, previously a Cre recombinase reporter assay for translocation (CRAfT) was established to determine the translocation of heterologous effector proteins using *E. coli* as recipient^8^. In this assay, TraJ, the T4CP of conjugation plasmid pKM101 T4SS, was remodeled by swapping the cytoplasmic domain of TraJ with the counterpart of VirD4 of T4SS in *A. tumefaciens*, *A. phagocytophilum*, or *Wolbachia pipientis* to create chimeric T4CP, TraJ-VirD4, in which the NTD of TraJ is kept for interaction with other VirBs of pKM101 T4SS. When co-expressed with Cre-tagged heterologous effector proteins in *E. coli*, cytoplasmic domain of TraJ-VirD4 recognizes its cognate substrates and delivers them to *E. coli* recipient cells through pKM101 T4SS. In recipient cells, Cre-tagged substrate proteins cleave *lox*P sites flanking the reporter gene, leading to the phenotypic change. By this assay, several T4SS effectors were confirmed, including Ats-1 of *A. phagocytophilum*. Followed the same rationale, we employed another broad-host-range plasmid RP4 and TEM-1 β-lactamase reporter to establish a more straightforward system for assaying the translocation of *A. phagocytophilum* effector proteins to eukaryotic recipient cells. Promiscuous plasmid RP4 can transfer substrates to a broad range of host cells including bacteria, fungi, and eukaryotic cells^32,33^. TEM-1 β-lactamase is widely used as reporter in applications such as mammalian gene expression assay, and bacterial effector protein translocation assay in eukaryotic cells^30,31^. To establish this assay system, we utilize a *E. coli* strain SM10 λpir as donor since it hosts integrated plasmid RP4^34^. Two types of plasmids were constructed, one expresses NTD of T4CP (TraG) of RP4 T4SS and cytoplasmic domain of VirD4 of *A. phagocytophilum* fusion protein (TraG-VirD4)^8,35^, the other expresses TEM-1. TraG-VirD4 gene was cloned into plasmid pMal-C5X under control of promoter *tac*. The ampicillin resistance gene, *bla* in pMal-C5X was then replaced with streptomycin resistance gene, *Sm*^*R*^ to avoid interference with TEM-1 in assay, leading to the generation of plasmid pTraG-VirD4 (Fig. 1A,C). TEM-1 β-lactamase gene expression cassette with promoter *tac* and transcription terminator *rrn*BT1 and T2 is cloned into plasmid pACYCDuet-1 for replacement of T7-based protein expression elements, leading to the generation of plasmids pTEM-1 and pTEM-1-X (X: *A. phagocytophilum* proteins) (Fig. 1B,D). For investigation of effector translocation, SM10 λpir expressing TraG-VirD4 and TEM-1 or TEM-1-X were incubated with Chinese hamster ovary K1 (CHO K1) cells, followed by the loading with CCF2-AM, a fluorescent substrate for β-lactamase. If TEM-1-X is transported into CHO K1 cells, it cleaves CCF2-AM, leading to emission of blue light, instead of green light, under fluorescent microscope. We first use a confirmed T4SS effector, Ats-1 to validate the system. CHO K1 cells incubated with SM10 λpir expressing TraG-VirD4 and TEM-1-Ats-1 have higher percent of blue cells than those incubated with SM10 λpir expressing TraG-VirD4 and TEM-1 (0.367 ± 0.012% vs. 0.040 ± 0.010%) (Fig. 2), suggesting that TEM-1-Ats-1 is transported into CHO K1 cells. To determine whether the translocation of TEM-1-Ats-1 is T4SS dependent, plasmid pSD_VirD4_ which expresses only cytoplasmic domain of VirD4, was constructed by deletion of DNA sequence encoding NTD of TraG in pTraG-VirD4. The NTD-deleted T4CP loses interaction with T4SS^9^, thereby it fails to translocate substrates. CHO K1 cells incubated with SM10 λpir expressing SD_VirD4_ and TEM-1-Ats-1 have lower percent of blue cells, compared to those incubated with SM10 λpir expressing TraG-VirD4 and TEM-1-Ats-1 (0.020 ± 0.010% vs. 0.367 ± 0.012%) (Fig. 2), suggesting the translocation of TEM-1-Ats-1 is T4SS dependent. Thus TEM-1 β-lactamase based protein translocation reporter system for *A. phagocytophilum* T4SS is established.

### Translocation of APH0215 into CHO K1 cells in TEM-1 β-lactamase based protein translocation assay

**Figure 3:**
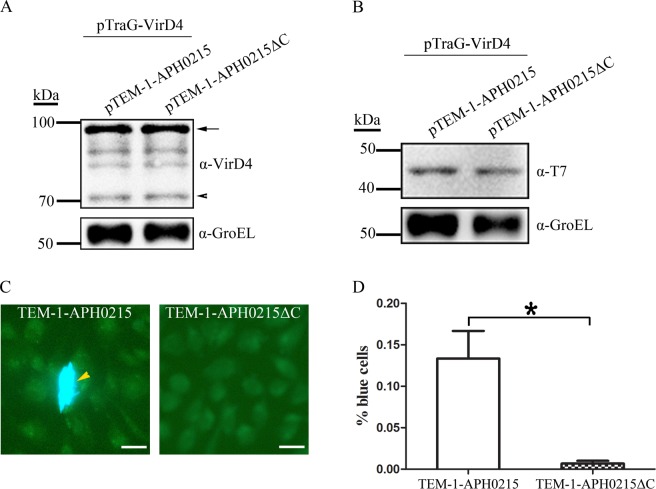
The essential role of C-terminus in the translocation of APH0215. (**A**) Western blot analysis for TraG-VirD4 expression, probed with rabbit anti-VirD4 antibody, in SM10 λpir cotransformed with plasmid pairs pTraG-VirD4 and pTEM-1-APH0215, and pTraG-VirD4 and pTEM-1-APH0215ΔC. Bacterial cultures were harvested after 2 h induction with IPTG, and before incubation with CHO K1 cells. Arrow indicates the bands with the expected size. Arrowhead indicates the degraded products of TraG-VirD4. GroEL bands, probed by anti-GroEL antibody were used to normalize the loading amount of bacterial lysates. (**B**) Western blot analysis for TEM-1-APH0215 and TEM-1-APH0215ΔC expression, probed with mouse monoclonal anti-T7 tag antibody, in SM10 λpir cotransformed with plasmid pairs pTraG-VirD4 and pTEM-1-APH0215, and pTraG-VirD4 and pTEM-1-APH0215ΔC. Bacterial cultures were harvested after 2 h induction with IPTG, and before incubation with CHO K1 cells. GroEL bands, probed by anti-GroEL antibody were used to normalize the loading amount of bacterial lysates. (**C**) Fluorescence signal conversion in CHO K1 cells incubated with SM10 λpir cotransformed with plasmid pairs pTraG-VirD4 and pTEM-1-APH0215 (TEM-1-APH0215), or pTraG-VirD4 and pTEM-1-APH0215ΔC (TEM-1-APH0215ΔC). A CHO K1 cell emitting blue fluorescence is highlighted with an arrowhead. Scale bars: 20 μm. (**D**) The percentage of blue cells in CHO K1 cells after incubation with SM10 λpir cotransformed with plasmid pairs pTraG-VirD4 and pTEM-1-APH0215 (TEM-1-APH0215), or pTraG-VirD4 and pTEM-1-APH0215ΔC (TEM-1-APH0215ΔC). *: Significant difference (*P* < 0.01) between groups indicated with line by Student *t*-test. Data are presented as the means and standard deviations of samples from three independent experiments. The full-length blots for all cropped ones are included in Supplemental Fig. S2 respectively.

There are common features in T4SS substrates, such as the positive charge at their C-termini, proteins with unknown functions (hypothetical proteins), limited similarities with proteins in other bacteria, and the presence of eukaryotic protein domains or protein-interacting motif in their amino acid sequences^31^. According to these features, bioinformatic approaches were employed to identify a multitude of T4SS effector proteins for several Gram-negative bacteria, such as *L. pneumophila*, *C. burnetii*, and *Brucella*^31,36,37^. More than 70 hypothetical proteins of *A. phagocytophilum* were identified bearing these features (data not shown). Among them APH0215 and APH0756 were chosen for assaying their translocation by this TEM-1 based system, since APH0215 has a eukaryotic protein domain, Sec24 (E value: 2.19e-07) and positively charged amino acids at its C-terminus (Fig. 2A), and APH0756 has positive charge at its C-terminus, and a coiled-coil motif. APH0215 and APH0756 genes were cloned into plasmid pTEM-1 to generate pTEM-1-APH0215 and pTEM-1-APH0756, respectively. SM10 λpir cells harboring plasmid pairs, pTEM-1 and pTraG-VirD4, pTEM-1-APH0215 and pTraG-VirD4, pTEM-1-APH0215 and pSD_VirD4_, pTEM-1-APH0756 and pTraG-VirD4, or pTEM-1-APH0756 and pSD_VirD4_, were subjected to the translocation assay. CHO K1 cells incubated with TEM-1-APH0215, TraG-VirD4-expressing SM10 λpir showed higher percent of blue cells than those incubated with TEM-1, TraG-VirD4-expressing SM10 λpir, or TEM-1-APH0215, SD_VirD4_-expressing SM10 λpir (0.167 ± 0.058% vs. 0.040 ± 0.010% vs. 0.020 ± 0.010%) (Fig. 2). However, the percentage of blue cells in CHO K1 cells incubated with TEM-1-APH0756, TraG-VirD4-expressing SM10 λpir does not showed significant difference from those incubated with TEM-1-APH0756, SD_VirD4_-expressing SM10 λpir (0.017 ± 0.006% vs. 0.013 ± 0.006%) (Fig. 2). These results suggest that APH0215, not APH0756 is transported into CHO K1 cells by RP4 T4SS, indicating APH0215 is a T4SS effector protein of *A. phagocytophilum*. The T4SS effector proteins have a positive charge at their C-termini, which plays an essential role in their translocation. To determine whether it is the case for APH0215, plasmid pTEM-1-APH0215ΔC was constructed, in which the DNA sequence encoding the C-terminal 21 amino acids of APH0215 (HRSHKCIITYLTDYSIGRSRT) was deleted. Compared to the pair, pTEM-1-APH0215ΔC and pTraG-VirD4, pTEM-1-APH0215 and pTraG-VirD4 pair produced more blue cells (0.133 ± 0.058% vs. 0.007 ± 0.006%), indicating that the positively charged C-terminus of APH0215 plays an important role in its translocation (Fig. 3).

### Subcellular localization of APH0215 in transfected cells

**Figure 4:**
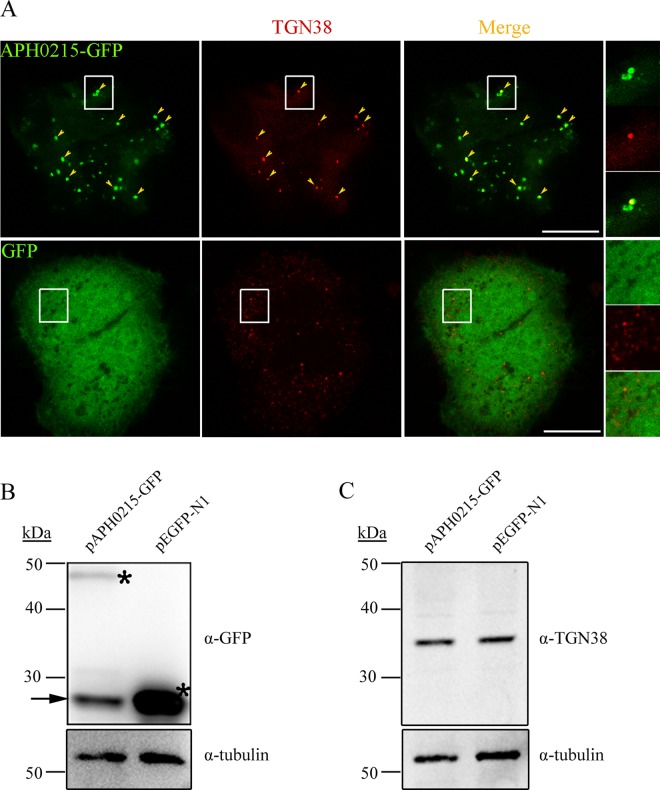
Colocalization of APH0215-GFP with *trans*-Golgi network. (**A**) HeLa cells transfected with plasmid expressing APH0215-GFP, or GFP (green color), were subjected to immunofluorescence labeling with mouse monoclonal antibody against TGN38, a *trans*-Golgi network protein (red color). Arrowheads indicate the colocalization between APH0215-GFP and TGN38. The boxed area are magnified on the right. Scale bars: 5 μm. (**B**) The expression of APH0215-GFP and GFP in transfected cells. HeLa cells transfected with plasmid expressing APH0215-GFP (pAPH0215-GFP), or GFP (pEGFP-N1), were subjected to Western blot analysis using mouse monoclonal antibodies against GFP and β-tubulin (tubulin). Asterisks highlight the expected bands. Arrow indicates the degraded product of APH0215-GFP. β-tubulin is used as internal control for sample loading. (**C**) The expression level of TGN38 in transfected cells. HeLa cells transfected with plasmid expressing APH0215-GFP (pAPH0215-GFP), or GFP (pEGFP-N1), were subjected to Western blot analysis using mouse monoclonal antibodies against TGN38 and β-tubulin (tubulin). β-tubulin is used as internal control for sample loading. The full-length blots for all cropped ones are included in Supplemental Fig. S3 respectively.

When replicating in infected cells, *A. phagocytophilum* intercepts vesicles from different sources to support its growth, such as those from autophagic pathway and *trans*-Golgi network, as indicated by the presence of autophagosome marker, LC3, and *trans*-Golgi network marker, TGN46/TGN38 inside of *A. phagocytophilum* inclusions^18,19,26^. APH0125 harbors eukaryotic protein domain, Sec24, which is an important component of coat protein complex II (COPII), promoting the formation of transport vesicles from the endoplasmic reticulum (ER) to Golgi apparatus. To determine the subcellular localization of APH0215, HeLa cells were transfected with plasmid pAPH0215-GFP and subjected to immunofluorescence labeling. APH0215-GFP was showed as punctuate pattern and majority of them were colocalized with TGN38 (Fig. 4), suggesting that APH0215-GFP vesicles interact with *trans*-Golgi network. Of note, compared to GFP expression, APH0215-GFP is expressed at lower level in transfected cells, and the TGN38 level does not show significant difference between APH0215-GFP-expressing cells and GFP-expressing cells (Fig. 4).

## Discussion

Bacterial T4SS plays an important role in the pathogenesis by dysregulating physiological processes of host cells through its transported effector proteins. Although a myriad of effector proteins was identified in intracellular bacteria, such as *L. pneumophila*, *C. burnetii*, and *Brucella*^31,36,37^, a very limited number of T4SS substrates was revealed in *A. phagocytophilum*. Most T4SS effector proteins bear typical signals, such as the positive charge at their C-termini. Based on these signals or features, T4SS effector proteins were initially identified by bioinformatic analysis, followed by the verification in effector protein translocation assay^31^. Several translocation assays were established based on Cre-*lox*P recombination, or the enzyme activities of the adenylate cyclase (Cya) domain of the *Bordetella pertussis* Cya toxin, and TEM-1 β-lactamase. Compared to Cre-*lox*P-based system which observes the gene transcription, and Cya-based system which measure intracellular cAMP level, TEM-1 β-lactamase offers a simple, flexible assay by observing the conversion of fluorescent substrate. Because of the difficulty in genetic manipulation of *A. phagocytophilum*, the verification of protein translocation was routinely performed in surrogate hosts including *A. tumefaciens*, *L. pneumophila*, *C. burnetii*, and recently *E. coli*. This newly developed Cre-*lox*P-based pKM101 conjugation system in *E. coli* has advantage over other systems since it uses T4CP for its cognate substrates as showed by demonstration of translocation of several rickettsial effector proteins, including *A. phagocytophilum* Ats-1 into *E. coli* recipient^8^. Although successfully used for identification of AnkA and HGE-14, two effectors of *A. phagocytophilum*, the coupling proteins of *A. tumefaciens* VirB/VirD4 and *L. pneumophila* Dot/Icm were not engineered, which might cause the lower translocation efficiency of *A. phagocytophilum* T4SS substrates compared to those in which the engineered coupling proteins are used, especially for *L. pneumophila* Dot/Icm, which belongs to phylogenetically distant T4BSS type. Meanwhile pKM101 conjugation system only transfers its substrates to bacterial recipient^8^, which prevents the use of TEM-1 β-lactamase as reporter in eukaryotic cells. In this study we take advantage of broad-host-range of RP4 conjugation plasmid to establish heterogeneous protein translocation assay using TEM-1 β-lactamase as reporter, and eukaryotic cells as recipient. Ats-1, a known effector protein was showed to be transported into CHO K1 cells in this assay, validating that this assay system can work with *A. phagocytophilum* T4SS effector proteins. We further applied this assay system to determine the translocation of two T4SS substrate feature*-*bearing *A. phagocytophilum* proteins. Sec24 domain-containing APH0215, not APH0756 was found to be transported into CHO K1 cells in this assay. Of note, due to the VirB protein difference between RP4 T4SS and *A. phagocytophilum* T4SS, this established protein translocation assay using chimeric coupling protein might not be suitable for all of *A. phagocytophilum* T4SS substrates. Sec24 is an important component of coat protein complex II (COPII) which promotes the formation of transport vesicles from ER^38^. It was reported that *A. phagocytophilum* extensively interacts with ER in vertebrate and invertebrate host cells, and recruits vesicles derived from ER and *trans*-Golgi network to *A. phagocytophilum*–containing compartments^21,26^. We found that APH0215 vesicles were colocalized with TGN38, suggesting there is the interaction between APH0215 and *trans*-Golgi network. However, whether APH0215 is involved in *A. phagocytophilum* interaction with ER and *trans*-Golgi network in *A. phagocytophilum* infection needs further investigation. In sum, we here developed a straightforward protein translocation assay for *A. phagocytophilum* T4SS, which will facilitate identification of *A. phagocytophilum* T4SS effector proteins and elucidation of HGA pathogenesis.

## Materials and Methods

### Construction plasmids pTraG-VirD4 and pTEM-1

To make chimeric coupling protein, TraG-VirD4 which is composed of the N-terminal transmembrane domain (NTD) of RP4 T4CP, TraG (1^st^–102^nd^ amino acids of TraG)^35^ and cytoplasmic domain of *A. phagocytophilum* VirD4 (103^rd^–740^th^ amino acids of VirD4)^8^, the DNA fragment encoding TraG-VirD4 was chemically synthesized (Talen-Bio Scientific Co, Shanghai, China) with two extra DNA sequences located at 5′- end (5′-*CAATTG*ACCAACAAGGACCATAGATTATG-3′; derived from plasmid pMal-c5X (New England Biolabs), containing MfeI endonuclease site and ribosomal binding site; Italicized nucleotides: MfeI site; Underline nucleotides: start codon) and 3′-end (5′-TAG*GGATCC*-3′; Underline nucleotides: stop codon; Italicized nucleotides: BamHI site) respectively. This DNA fragment was digested with MfeI and BamHI, followed by ligation with MfeI, BamHI-digested pMal-c5X and transformation into competent *E. coli* DH5α strain (Vazyme Biotech Co, Nanjing, China), leading to the generation of plasmid pTraG-VirD4-bla, in which original *malE* gene is replaced by *traG-virD4*, and the β-lactamase gene *bla* is still kept. Since β-lactamase expressed from pTraG-VirD4-bla will interfere with TEM-1 expressed from another plasmid, its gene is replaced by streptomycin resistance gene (Sm^R^). DNA fragment including coding region and promoter region of Sm^R^ was amplified by PCR using a pair of primers (SM-F and SM-R) (Table S1), and plasmid pCDFDuet-1 (Novagen) as template. This DNA fragment was digested with NdeI and NotI, and ligated with NdeI, NotI-digested PCR product amplified using pTraG-VirD4-bla as template, and a pair of primers (pMal-F and pMal-R) (Table S1). The ligated product was transformed into *E. coli* DH5α, leading to the generation of plasmid pTraG-VirD4-Sm^R^ (pTraG-VirD4), in which original β-lactamase gene *bla* is replaced by Sm^R^ gene. To make plasmid pVirD4_,_ which only expresses the cytoplasmic domain of VirD4 of *A. phagocytophilum*, the PCR product amplified using a pair of primers (VirD4-F and VirD4-R) (Table S1) and pTraG-VirD4 as template, was digested with SalI and self-ligated, followed by transformation into *E. coli* DH5α, leading to the generation of pSD_VirD4,_ in which the coding region of NTD of RP4 TraG was deleted.

To construct plasmid that expresses TEM-1 β-lactamase reporter, DNA fragment containing pMal-c5X-derived promoter tac and transcription terminators *rrnB* T1 and T2, 6x His and bacteriophage T7 tag coding sequence, TEM-1 coding sequence, and multiple cloning sites (MCS) was commercially synthesized (Talen-Bio Scientific Co, Shanghai, China). By recombination-based cloning using ClonExpress II One Step Cloning Kit (Vazyme), the PCR product amplified using this synthesized DNA fragment as template and a pair of primers (TEM-1-F and TEM-1-R) (Table S1) was cloned into pACYCDuet-1 (Novagen) linearized by PCR using a pair of primers (pACYCDuet-F and pACYCDuet-R) (Table S1), leading to the generation of plasmid pTEM-1, in which original phage T7-based expression elements is replaced by ptac-based expression system for expressing TEM-1 and TEM-1-X fusion proteins. For constructing plasmid which expresses TEM-1-Ats-1, TEM-1-APH0215, or TEM-1-APH0756, DNA fragments encoding full length of Ats-1, APH0215, and APH0756 were amplified by PCR using *A. phagocytophilum* genomic DNA as template and responding primer pairs (Table S1), and cloned by using ClonExpress II One Step Cloning Kit into pTEM-1 linearized by NdeI digestion, leading to the generation of plasmids pTEM-1-Ats-1, pTEM-1-APH0215, and pTEM-1-APH0756. To construct pTEM-1-APH0215ΔC, in which the DNA sequence encoding the C-terminal 21 amino acids of APH0215 was deleted, the PCR was performed using a pair of primers (215-Delta-C-R and 215-Delta-C-F) (Table S1) and pTEM-1-APH0215 as template. The PCR product was digested with BamHI and self-ligated, leading to the generation of pTEM-1-APH0215ΔC.

After the plasmids were purified and sequenced, plasmid pairs were cotransformed into *E. coli* SM10 λpir strain (*Km*^*R*^*, thi-1, thr, leu, tonA, lacY, supE, recA*::RP4-2-Tc::Mu, pir)^34^ using the method published elsewhere^39^.

For easy view, plasmid map of pTraG-VirD4 and pTEM-1 were made with the application SnapGene (GSL Biotech, Chicago, IL,USA) after their sequences were assembled.

### Protein translocation assay

CHO K1 cells (Invitrogen) were propagated in F-12K medium (Gibco) supplemented with 10% fetal bovine serum (Gibco). Cultures were incubated at 37 °C in a humidified 5% CO_2_/95% air atmosphere. The day before the assay, the CHO K1 cells were split onto cover slips in 24-well plate with 5 × 10^4^ cells/well. On the day of assay, SM10 λpir harboring responding plasmids were cultured in Luria–Bertani (LB) medium containing spectinomycin (50 μg/mL) and chloramphenicol (50 μg/mL) to 0.3 OD_600_, followed by the addition of Isopropyl β-D-1-thiogalactopyranoside (IPTG) to final concentration of 1 mM to induce protein expression. After 2 h induction, 1 mL of SM10 λpir cultures were assigned to Western blot analysis to determine protein expression. The remaining cultures were centrifuged at 5000 × *g* for 5 min, and the bacterial pellets were resuspended with the same volume of completed F-12K medium supplemented with 1 mM IPTG. CHO K1 cells with 80% confluence were washed with 1 x PBS (137 mM NaCl, 2.7 mM KCl, 10 mM Na_2_HPO4, and 2 mM KH_2_PO4, pH 7.4), followed by the addition with SM10 λpir suspension at 0.5 mL/well (ratio of bacteria/cells is 200:1) and centrifugation at 250 × *g* for 10 min. After CHO K1 cells were incubated with SM10 λpir for additional 2 h, the cells were washed by PBS for 3 times to remove bacteria, followed by the loading of fluorescent substrate CCF2-AM in the LiveBLAzer-FRET B/G Loading Kit (Invitrogen), performed according to manufacturer’s instruction. The loading of CCF2-AM into CHO K1 cells took 90 min at room temperature (RT), protecting from light. After washing with PBS, CHO K1 cells were fixed with 4% paraformaldehyde solution for 20 min at RT, followed by the observation under Nikon Eclipse Ni fluorescence microscope (Nikon, Tokyo, Japan) equipped with Chroma filter set (Cat#:19011; Excitation filter: AT405/30× , Emission filter: AT450lp, and Dichroic mirror: AT440DC). To determine the conversion of green to blue light, % blue cells in at least 1000 CHO K1 cells from each group in three independent experiments was calculated.

### Expression and purification of recombinant truncated *A. phagocytophilum* VirD4, and preparation of rabbit anti-VirD4 sera

Using *A. phagocytophilum* genomic DNA as template, DNA fragment encoding truncated *A. phagocytophilum* VirD4 (191^st^–740^th^ amino acids) was amplified by PCR with a pair of primers (VirD4-Expression-F and VirD4-Expression-R) (Table S1). For protein expression, the PCR products were digested with EcoRI and NotI, ligated with pET28a (+) (prokaryotic expression vector, Novagen), and transformed into *E. coli* BL21 (DE3) (Vazyme). Recombinant *E. coli* BL21 (DE3) were cultured in LB medium containing kanamycin (34 μg/mL) at 37 °C with shaking at 250 rpm. After bacteria were cultured to reach the mid-logarithmic phase of growth (OD_600_ value of 0.4), IPTG was added at the final concentration of 1 mM to induce recombinant protein expression. After induction at 37 °C for 4 h, bacterial cultures were centrifuged at 10 000 × *g* for 10 min at 4 °C. The pellet was lysed and recombinant protein was affinity-purified with Ni-NTA resin (Qiagen) under denaturing condition as instructed by manufacturer. Purified recombinant protein was subjected to commercial antibody production in rabbit host (Abgent Biotech, Suzhou, China).

### Transfection and immunofluorescence labeling

To determine the cellular localization of APH0215 in transfected cells, the plasmid pAPH0215-GFP was constructed and transfected into HeLa cells, followed by immunofluorescence labeling. Briefly, DNA fragment encoding full-length APH0215 was amplified by PCR using a pair of primers (APH0215-GFP-F and APH0215-GFP-R) (Table S1) and *A. phagocytophilum* genomic DNA as template, followed by digestion with XhoI and BamHI, and ligation with eukaryotic expression vector pEGFP-N1 (Clontech), leading to the generation of plasmid pAPH0215-GFP. pAPH0215-GFP and pEGFP-N1 were then transfected into HeLa cells by Polyethylenimine MAX in EZ Trans transfection kit according to manufacturer’s instruction (Shanghai Life iLab Biotech Co, Shanghai, China), respectively. For IFA, the transfected cells were harvested at 36 h post-transfection, and subjected to fixation with 2% paraformaldehyde in PBS at RT for 30 min and washing with PBS for three times, followed by permeabilization and blocking with PS solution (PBS containing 0.8% BSA and 0.3% saponin) at RT for 10 min. Immunofluorescence labeling was performed using mouse monoclonal antibody against TGN38 (Clone B-6, Santa Cruz Biotechnology) and Alexa Fluor 555-conjugated goat anti-mouse IgG (Invitrogen). Cells were observed under Leica TCS SP8 confocal microscope (Leica).

### Western blot analysis

SM10 λpir and SM10 λpir harboring plasmids were centrifuged at 10, 000 × *g* for 1 min after induced with 1 mM IPTG for 2 h in the protein translocation assay. Bacterial pellet from 1 mL culture was dissolved in 100 μl of 2 × SDS-PAGE loading buffer (4% SDS, 135 mM Tris–HCl [pH 6.8], 10% glycerol, and 10% β-mercaptoethanol). Samples were separated by SDS-PAGE with 12% polyacrylamide resolving gels, then transferred to nitrocellulose membrane using mini Trans-blot cell (Bio-Rad). The membrane blocking was performed with blocking buffer (5% (wt/vol) skim milk (BD Difco) in PBS buffer) for 30 min at RT, followed by incubation with mouse monoclonal anti-T7 antibody (Millipore, diluted in blocking buffer at ratio 1:5000), rabbit anti-VirD4 antibody (diluted in blocking buffer at ratio 1:2000), or mouse monoclonal anti-*E. coli* GroEL antibody (abcam, diluted in blocking buffer at ratio 1:5000) at RT for 1 h. After washing for three times with PBS (10 min each time), the membranes were probed with peroxidase-conjugated goat anti-mouse IgG, or anti-rabbit IgG secondary antibody (KPL, Gaithersburg, MD, diluted in blocking buffer at ratio 1:5000) at RT for 1 h. The membranes were washed four times with PBS (10 min each time), followed by ECL chemiluminescence. The images were captured by Tanon 4200 chemiluminescent imaging system (Tanon, Shanghai, China). Similarly, to determine the expression of APH0215-GFP, GFP, and TGN38 in transfected cells, HeLa cells transfected with pAPH0215-GFP or pEGFP-N1 were harvested at 36 h post-transfection, and subjected to probing with mouse monoclonal antibodies against GFP (TransGen Biotech, Beijing, China), TGN38, and β-tubulin (Beijing CoWin Biotech Co) in Western blot analysis. *E. coli* GroEL and human β-tubulin were used as internal controls for sample loading. Due to the abundance of GroEL in *E. coli* and GFP in transfected cells, which shadows other weak bands in image capture by CCD camera, sequential incubation with antibodies were performed. To determine the expression VirD4 and TEM-1 expression in SM10 λpir, nitrocellulose membranes were first probed with anti-VirD4 antibody or anti-T7 tag antibody, and developed, followed by incubation with anti-GroEL antibody and development. For determination of APH0215-GFP and GFP in transfected cells, nitrocellulose membranes were sequentially incubated with antibodies against β-tubulin and GFP. For determination of TGN38 in pAPH0215-GFP or pEGFP-N1-transfected cells, nitrocellulose membranes were concurrently incubated with antibodies against TGN38 and β-tubulin.

### GenBank accession number

*A. phagocytophilum* VirD4: ABD44318; APH0215: ABD44037; APH0756: ABD44018.

### Statistical analysis

Three independent experiments were conducted and data were expressed as mean ± S.D. Statistical significance was evaluated by the Student *t*-test.

The materials and data generated in the current study are available from the corresponding author on reasonable request.

## Supplementary information

Supplementary information
