# Supplementary material for: Development of TEM-1 β-lactamase based protein translocation assay for identification of Anaplasma phagocytophilum type IV secretion system effector proteins
Source: Sci Rep. 2019 Mar 12;9:4235. doi: 10.1038/s41598-019-40682-8 (PMC6414681; doi:10.1038/s41598-019-40682-8)

Development of TEM-1 β-lactamase based protein translocation assay for identification of *Anaplasma phagocytophilum* type IV secretion system effector proteins

Jiafeng Zhu, Meiling He, Wenting Xu, Yuanyuan Li, Rui Huang, Shuyan Wu, Hua Niu*

Department of Microbiology, College of Medicine, Soochow University, Suzhou, Jiangsu Province, 215123, China.

*Corresponding Author:
Hua Niu, PhD
Department of Microbiology, College of Medicine, Soochow University,
199 Ren Ai Road,
Suzhou, Jiangsu Province, 215123, China.
E-mail: hniu@suda.edu.cn, or niu_hua@126.com
Tel: +86-512-65880132

Supplementary Information

Table S1. Primers used for PCR amplification

Primer ID	Sequence (5'-3')	Note	Purpose
SM-F	CTTGCGGCCGCCTGCTTATCCACGGAATCT	NotI site is underlined	Replacement of ampicillin resistance gene *bla* with Sm^R in plasmid pTraG-VirD4
SM-R	TCACATATGTTATTTGCCGACTACCTTGGTGATC	NdeI site is underlined	
pMal-F	TCACATATGCTGTCAGACCAAGTTTACTCAT	NdeI site is underlined	
pMal-R	CTTGCGGCCGCAACGGACCGAAAGAGTTT	NotI site is underlined	
VirD4-F	TCCGTCGACCGACATGTTCTTATTGATTGGC	SalI site is underlined	Construction of pVirD4
VirD4-R	TCCGTCGACCATAATCTATGGTCCTTGTTGG	SalI site is underlined	
TEM-1-F	TATGCGACTCCTGCATTAGGCATAACG		Cloning of TEM-1 β-lactamase gene into plasmid pACYCDuet-1 to generate plasmid pTEM-1
TEM-1-R	TGCTTCTCAAATGCCTGAGGAGTTTGTAGAAAC		
pACYCDuet-F	CCTCAGGCATTTGAGAAGCACA		
pACYCDuet-R	CCTAATGCAGGAGTCGCATAAGG		
TEM-1-Ats-1-F	GGCGGTGGAGGCAGCCATATGCTAATAAGAAGAATTCTGAC		Construction of plasmid pTEM-1-Ats-1
TEM-1-Ats-1-R	GCGGCCGCCCATGGACATATGTTACCTCGTACCTTTACCAT		
TEM-1-APH0215-F	GGCGGTGGAGGCAGCCATATGGTCGATAACACCACGAT		Construction of plasmid pTEM-1-APH0215
TEM-1-APH0215-R	GCGGCCGCCCATGGACATATGTTACGTTCTCGATCGGCC		
TEM-1-APH0756-F	GGCGGTGGAGGCAGCCATATGCGTCCTGATGGGCAAC		Construction of plasmid pTEM-1-APH0756
TEM-1-APH0756-R	GCGGCCGCCCATGGACATATGTTATCGCGAGACACCACTTC		
VirD4-Expression-F	ATCCGAATTCAAGCTTGAAAACTACGACCTCAC	EcoRI site is underlined	Expression of truncated recombinant VirD4 protein
VirD4-Expression-R	CTTGCGGCCGCCTTTAGTCTTCCGTTACTGCTATC	NotI site is underlined	
215-Delta-C-R	TAGGGATCCTTAATCGCAGTGTATGTCATAAAAAGT	BamHI site is underlined	Construction of plasmid pTEM-1-APH0215ΔC
215-Delta-C-F	ATCGGATCCGAATTCCCTGCA	BamHI site is underlined	

APH0215-GFP-F	GCA<u>CTCGAG</u>GCCACCATGGTCGA TAACACCACGATAC	XhoI site is underlined	Construction of plasmid pAPH0215-GFP
APH0215-GFP-R	GGT<u>GGATCC</u>CGCGTTCTCGATCGG CCAATAG	BamHI site is underlined	

Supplemental Fig. S1

Full-length blots for Figure 2B and 2C.

Supplemental Fig. S1

Zhu et al.

Relative to Fig. 2B

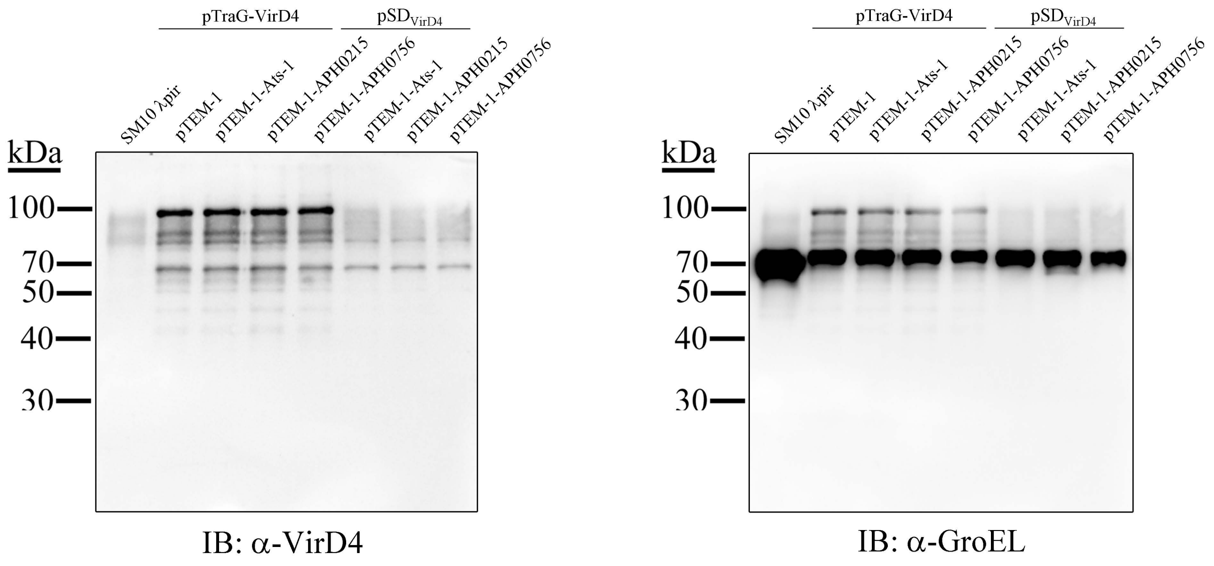

Relative to Fig. 2C

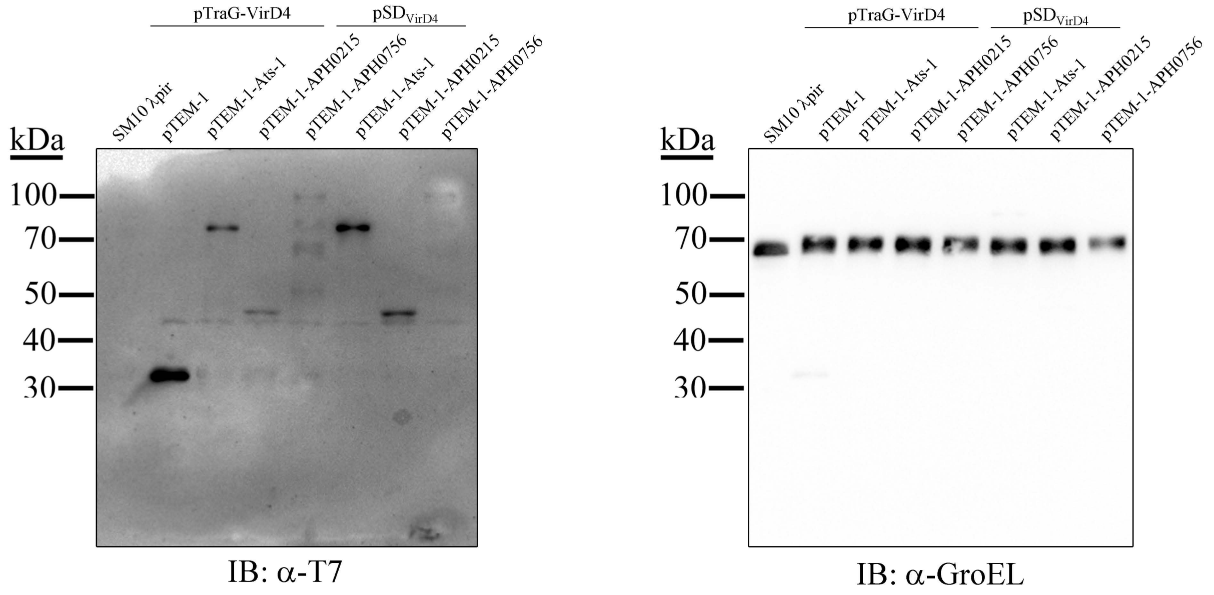

Supplemental Fig. S2

Full-length blots for Figure 3A and 3B.

Supplemental Fig. S2

Zhu et al.

Relative to Fig. 3A

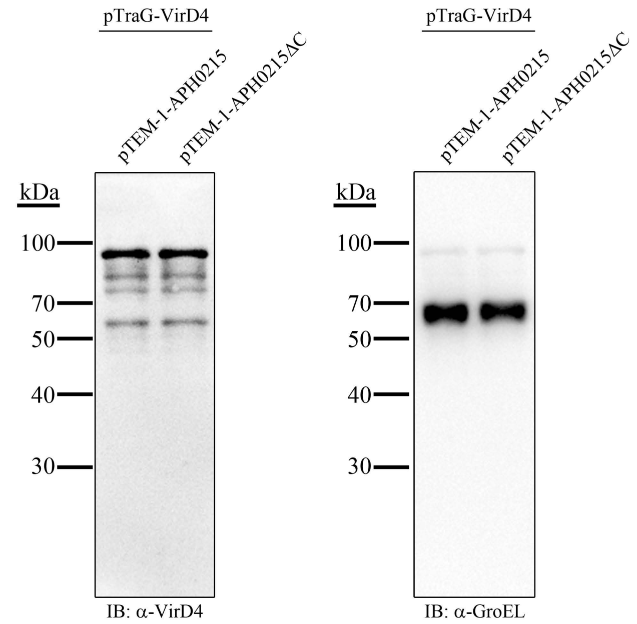

Relative to Fig. 3B

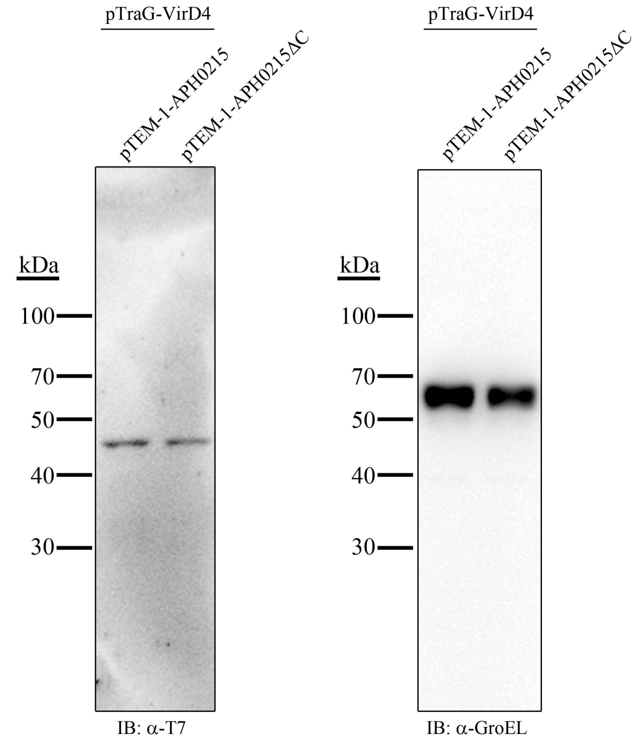

Supplemental Fig. S3

Full-length blots for Figure 4B and 4C.

Supplemental Fig. S3

Zhu et al.

Relative to Fig. 4B

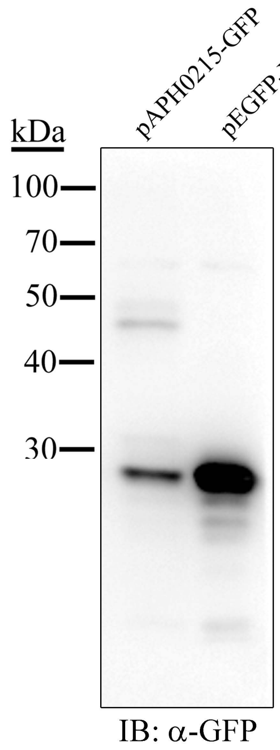

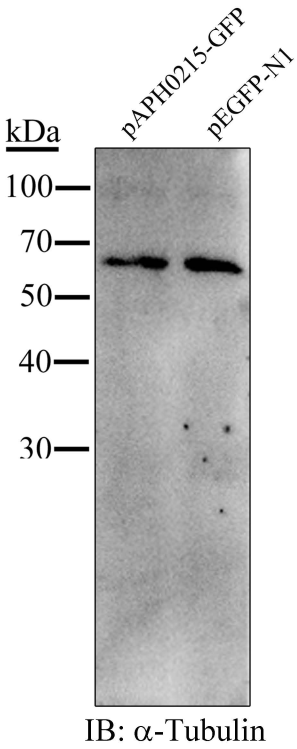

Relative to Fig. 4C

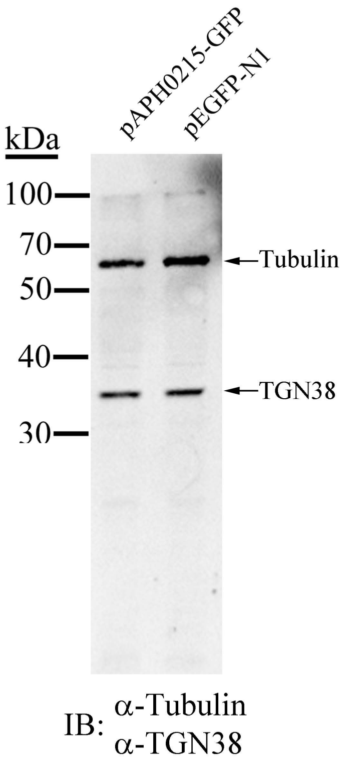

Supplement: Supplementary file 1 — Supplementary information [file 41598_2019_40682_MOESM1_ESM.pdf]
